# An epigenome-wide association study of sex-specific chronological ageing

**DOI:** 10.1186/s13073-019-0693-z

**Published:** 2019-12-31

**Authors:** Daniel L. McCartney, Futao Zhang, Robert F. Hillary, Qian Zhang, Anna J. Stevenson, Rosie M. Walker, Mairead L. Bermingham, Thibaud Boutin, Stewart W. Morris, Archie Campbell, Alison D. Murray, Heather C. Whalley, David J. Porteous, Caroline Hayward, Kathryn L. Evans, Tamir Chandra, Ian J. Deary, Andrew M. McIntosh, Jian Yang, Peter M. Visscher, Allan F. McRae, Riccardo E. Marioni

**Affiliations:** 1 Centre for Genomic and Experimental Medicine, Institute of Genetics and Molecular Medicine, University of Edinburgh, Edinburgh, Scotland, UK; 2 Institute for Molecular Bioscience, University of Queensland, Brisbane, QLD Australia; 3 Centre for Cognitive Ageing and Cognitive Epidemiology, University of Edinburgh, Edinburgh, Scotland, UK; 4 MRC Human Genetics Unit, Institute of Genetics and Molecular Medicine, University of Edinburgh, Edinburgh, Scotland, UK; 5 Aberdeen Biomedical Imaging Centre, University of Aberdeen, Aberdeen, Scotland, UK; 6 Division of Psychiatry, Royal Edinburgh Hospital, University of Edinburgh, Edinburgh, Scotland, UK; 7 Department of Psychology, University of Edinburgh, Edinburgh, Scotland, UK; 8 Institute for Advanced Research, Wenzhou Medical University, Wenzhou, Zhejiang, 325027 China

**Keywords:** DNA methylation, Ageing, Sexual dimorphism, X chromosome, Generation Scotland

## Abstract

**Background:**

Advanced age is associated with cognitive and physical decline and is a major risk factor for a multitude of disorders. There is also a gap in life expectancy between males and females. DNA methylation differences have been shown to be associated with both age and sex. Here, we investigate age-by-sex differences in blood-based DNA methylation in an unrelated cohort of 2586 individuals between the ages of 18 and 87 years, with replication in a further 4450 individuals between the ages of 18 and 93 years.

**Methods:**

Linear regression models were applied, with stringent genome-wide significance thresholds (*p* < 3.6 × 10^−8^) used in both the discovery and replication data. A second, highly conservative mixed linear model method that better controls the false-positive rate was also applied, using the same genome-wide significance thresholds.

**Results:**

Using the linear regression method, 52 autosomal and 597 X-linked CpG sites, mapping to 251 unique genes, replicated with concordant effect size directions in the age-by-sex interaction analysis. The site with the greatest difference mapped to *GAGE10*, an X-linked gene. Here, DNA methylation levels remained stable across the male adult age range (DNA methylation by age *r* = 0.02) but decreased across female adult age range (DNA methylation by age *r* = − 0.61). One site (cg23722529) with a significant age-by-sex interaction also had a quantitative trait locus (rs17321482) that is a genome-wide significant variant for prostate cancer. The mixed linear model method identified 11 CpG sites associated with the age-by-sex interaction.

**Conclusion:**

The majority of differences in age-associated DNA methylation trajectories between sexes are present on the X chromosome. Several of these differences occur within genes that have been implicated in sexually dimorphic traits.

## Background

Advanced age is associated with cognitive and physical decline and is a major risk factor for a multitude of disorders including cancer, cardiovascular disease, and neurodegenerative diseases. Furthermore, males and females, on average, exhibit disparate risk profiles for various disease states as well as different life expectancies [1, 2]. Indeed, the average life expectancy at birth in Scotland is 77.0 years for males and 81.1 years for females as reported by the Life Tables for Scotland 2015–2017 [3]. Thus, markers of ageing are merited, including those which can exploit sexual dimorphism for sex-specific prediction of ageing and disease risk. Biological hallmarks of ageing have been observed at the cellular and molecular level and include shortening of telomeres, genomic instability, and both global and local changes in DNA methylation (DNAm) levels [4–6]. DNAm is a common epigenetic mark, typically occurring in the context of a cytosine-guanine dinucleotide motif (CpG). It can be modulated by both environmental exposures and genetic variation and is sexually dimorphic [7].

Elastic net regression and best linear unbiased predictor models have been used to robustly predict chronological age by leveraging inter-individual variation in methylation profiles [8–10]. These methodologies capture global-level DNAm changes with respect to ageing but fail to inform the contribution of individual loci to the ageing process. These biological age predictors, also referred to as ‘epigenetic clocks’, correlate strongly with chronological age. Additionally, for a given chronological age, an advanced epigenetic age is associated with increased mortality risk and many age-related morbidities [11]. Importantly, males exhibit increased DNAm-based age acceleration relative to females (i.e. a ‘faster ticking’ epigenetic clock) supporting the role of epigenetic perturbations in sex-specific ageing trajectories [8, 9].

In 2013, Horvath proposed a pan-tissue epigenetic clock derived from the linear combination of 353 CpGs whereas Hannum et al. created a DNAm-based clock based on 71 CpGs in blood tissue [8, 9]. Following on from these seminal studies, Zhang et al. developed a highly precise DNAm-based predictor of chronological age (ZhangAge) limiting the value of such estimators as biomarkers of ageing [10]. Subsequently, a new generation of DNAm-based measures of ageing was proposed. Recently, Levine et al. proposed a powerful predictor of lifespan and health by developing a methylation-based predictor of an individual’s ‘phenotypic age’ (DNAm PhenoAge) [12]. Phenotypic age is informed by chronological age and physical and biochemical measures, such as albumin and mean cell volume. Finally, a novel clock, termed DNAm GrimAge, was trained using mortality as a reference and supplants predecessor clocks in predicting the risk of mortality and a number of age-related morbidities [13].

While global information relating to DNAm perturbations has been harnessed to measure biological ageing, little is known regarding the role of specific epigenetic loci in the ageing process. Presently, age-related hypermethylation at the *ELOVL2* locus on chromosome 6 remains the strongest known site-specific DNAm alteration throughout the lifespan [14]. Methylation status at *FHL2*, *KFL14*, *C1orf132*, and *TRIM59* has also been shown to exhibit linear associations with chronological age [15]. These data may highlight biologically important epigenetic substrates of the human ageing process. However, the loci which are differentially methylated between males and females, in the context of ageing, merit elucidation. Therefore, in the current study, we sought to identify sex differences in age-associated genome-wide DNAm changes, in the whole blood from a discovery sample of 2586 unrelated individuals. These were replicated in a further 4450 unrelated individuals. Both discovery and replication sets are derived from the same parent cohort: Generation Scotland [16]. The replication cohort was unrelated to the discovery cohort. Further understanding of the sex-specific effects on biological ageing, through the identification of differentially methylated loci, may assist in identifying novel risk factors for age- and sex-associated pathologies.

## Methods

### Generation Scotland: Scottish Family Health Study

Data came from the family-based Generation Scotland: Scottish Family Health Study (GS). GS participants were recruited from GP practices in 5 regions across Scotland between the years 2006 and 2011 [16]. The probands were aged between 35 and 65 years and were asked to invite first-degree relatives to join the study, which had a final size of 24,090. A variety of cognitive, physical, and health data were collected at the study baseline along with the blood or saliva samples for DNA genotyping. Blood-based DNAm data were obtained on a subset of 5200 participants using the Illumina EPIC array [17]. Quality control details have been reported previously [17]. Briefly, probes were removed based on (i) outliers from visual inspection of the log median intensity of the methylated versus unmethylated signal per array, (ii) a bead count < 3 in more than 5% of samples, and (iii) ≥ 5% of samples having a detection *p* value > 0.05. Samples were removed (i) if there was a mismatch between their predicted sex and recorded sex and/or (ii) if ≥ 1% of CpGs had a detection *p* value > 0.05. For the present analyses, we considered unrelated individuals from the DNAm subset of GS. A genetic relationship matrix was built using GCTA-GRM, and a relatedness coefficient of < 0.025 was specified to exclude related individuals [18]. In cases where a couple was present, 1 individual was removed to minimise shared environment effects. This left an analysis sample of 2586 unrelated individuals ranging in age from 18 to 87 years and 807,857 probes.

The second set of blood-based DNA methylation from Generation Scotland was released in early 2019 and was treated as a replication sample. This comprised 4450 individuals who were unrelated (genetic relatedness < 0.05) to each other and to the 5200 participants from the first Generation Scotland methylation data set. Quality control steps have been reported previously [19] and were near identical to those reported above.

### Statistical analysis

All analyses were performed in R version 3.5.3 [20].

### Epigenome-wide association studies

Epigenome-wide association studies (EWASs) of chronological age, sex, and the interaction between age and sex were performed using two approaches.

First, we considered linear regression models adjusted for smoking status (smoking pack-years and status—current, gave up in the last year, gave up more than a year ago, never, or unknown), estimated white blood cell proportions (CD8+ T cells, CD4+ T cells, natural killer cells, B cells, and granulocytes), methylation batch, and 20 methylation-based principal components to correct for unmeasured confounders. Age was centred by its mean, and sex was included as a factor. The models were run using the limma package in R (empirical Bayes moderated *t*-statistics) [21].

To remove the widespread effect of sex on X-linked methylation, we also ran sex-stratified age EWASs on the X chromosome. We compared this output with the results from the age-by-sex interaction model by plotting the –log_10_ *p* values from the interaction model against the –log_10_ *p* value from a heterogeneity test of the effects between the sex-stratified model: *χ*^2^_hetero_ = (beta_male_ − beta_female_)^2^/(SE_male_^2^ + SE_female_^2^).

Second, we considered the MOMENT method from the OmicS-data-based Complex trait Analysis software (OSCA) [22]. MOMENT is a mixed linear model-based method that can account for unobserved confounders and the correlation between distal probes which may be introduced by such confounders. Initially, a linear regression step is performed to obtain an association *p* value for each CpG site. The sites with *p* < 0.05/*n*_probes_ are included in the first component with the remainder feeding into a second component. These components are then fitted as random effects in the model to control for confounding. However, convergence issues can occur when a large number of CpGs are included in the first component, as is the case with EWASs of age and sex. To account for this, we implemented a stepwise selection procedure to avoid saturation of the first component (MOMENT2; http://cnsgenomics.com/software/osca/#EWAS). In the age EWAS models, we pre-adjusted the methylation data for sex and batch. Additional adjustment for cell counts and smoking resulted in genomic deflation due to collinearity between these covariates and age. In the sex EWAS models, we pre-adjusted the methylation data for age and batch. For the age-by-sex interaction EWAS models, the methylation data were pre-adjusted for age, sex, smoking status, smoking pack-year, cell types, and batch, and the age-by-sex interaction outcome was adjusted for age and sex. A default threshold (*p* < 0.05/*n*_probes_) was used to select probes for the first component, prior to the stepwise analysis, for all models. The only exception was the sex EWAS in the replication cohort where this threshold was increased to *p* < 1 × 10^−20^ to enable model convergence. Probes with a *p* value less than 3.6 × 10^−8^ [23] were considered epigenome-wide significant associations.

### Pathway analysis

Enrichment was assessed among the KEGG pathways and Gene Ontology (GO) terms using the gometh() function in the missMethyl package in R [24]. This function models the relationship between the number of probes per gene and the probability of being selected, accounting for the selection bias associated with probe-dense genes.

## Results

### Sample demographics

The genetically unrelated subset of Generation Scotland (discovery cohort) had a mean age of 50 years (SD = 12.5) and comprised 1587 females (61.4%) and 999 males (38.6%). Males ranged in age from 18.1 to 85.7 years (mean = 50.8 years, SD = 12.2) whereas females ranged from 18.0 to 86.9 years (mean = 49.5 years, SD = 12.7). The replication cohort had a mean age of 51.4 years (SD = 13.2) and comprised 2506 females (56.3%) and 1944 males (43.7%). Males ranged in age from 18.1 to 86.5 years (mean = 52.0 years, SD = 13.3) whereas females ranged from 18.1 years to 93.3 years (mean = 50.9 years, SD = 13.1).

### DNAm and chronological age

**Fig. 1:**
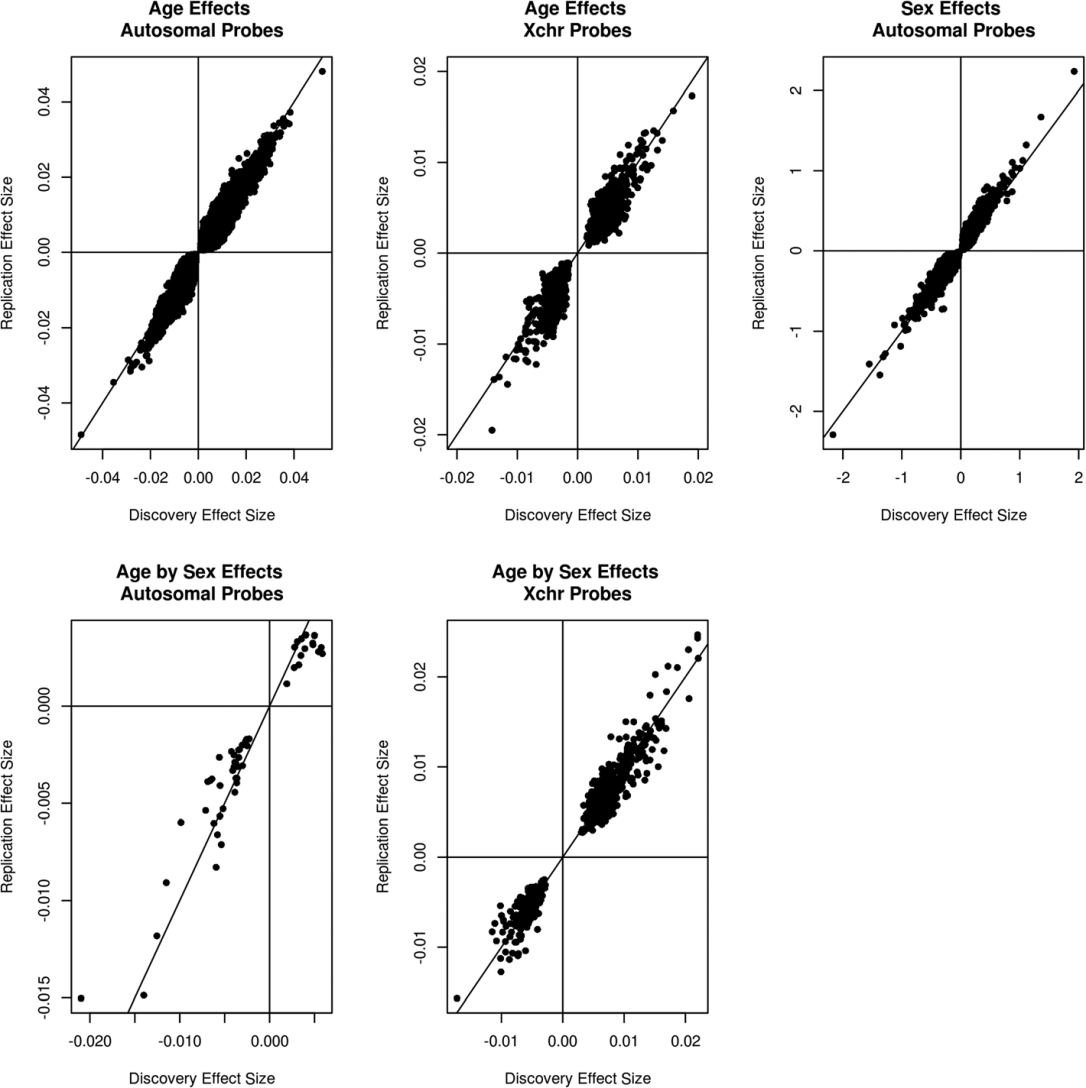
Age, sex, and age-by-sex effects in discovery and replication cohorts (linear regression method). Discovery cohort effect sizes (*x*-axis) are plotted against replication cohort effect sizes (*y*-axis) for the epigenome-wide analysis of age, sex, and age-by-sex. Autosomal and X-chromosome associations are presented separately

Using a linear regression (LR) model approach that did not adjust for inter-probe correlations, there were 250,485 autosomal and 3096 X-linked CpGs associated with chronological age at the epigenome-wide significant level (*p* < 3.6 × 10^−8^) in the discovery cohort. 151,537 autosomal and 1668 X-linked sites were epigenome-wide significant with the effect sizes in the same direction in the replication cohort (Fig. 1; Additional file 1: Tables S1-S2); 68,038 of these CpGs showed increasing methylation with age. High genomic inflation was observed with lambda values ranging from 12.2 to 57.1 (Additional file 2: Figure. S1).

A mixed linear model (MLM) approach that adjusted for inter-probe correlations and unmeasured confounders showed much better control of genomic inflation (lambda values ranging from 0.96 to 1.03; Additional file 2: Figure. S2). It identified 6 autosomal and 5 X-linked sites that were epigenome-wide significant with the effect sizes in the same direction in both the discovery and replication cohorts (Table 1). All of these sites were included in the 153,205 sites reported in Fig. 1 and Additional file 1: Tables S1-S2.

**Table 1:** Age, sex, and age-by-sex associations common to discovery and replication cohorts using both linear regression and MOMENT methods. The results shown correspond to the MOMENT output

Probe	Chr	BP	Gene	Orientation	Discovery b	Discovery se	Discovery p	Replication b	Replication se	Replication p	Phenotype
cg08097417	7	130,419,133	*KLF14*	–	4.26	0.40	5E−27	2.45	0.23	7E−27	Age
cg16867657	6	11,044,877	*ELOVL2*	+	5.05	0.40	4E−36	4.85	0.27	4E−71	Age
cg10501210	1	207,997,020	NA	+	− 1.90	0.19	3E−23	− 1.20	0.12	4E−23	Age
cg07553761	3	160,167,977	*TRIM59*	+	1.91	0.33	4E−09	1.25	0.20	1E−10	Age
cg23606718	2	131,513,927	*FAM123C*	+	2.45	0.32	1E−14	1.83	0.23	1E−15	Age
cg02872546	2	109,741,578	NA	–	− 3.63	0.51	6E−13	− 1.94	0.33	2E−09	Age
cg20351734	X	73,571,783	NA	–	− 6.90	0.63	4E−28	− 5.81	0.44	4E−39	Age
cg15833111	X	49,166,019	*GAGE10*	+	− 3.60	0.46	3E−15	− 2.41	0.26	3E−21	Age
cg25140188	X	31,087,348	NA	+	− 1.81	0.29	6E−10	− 1.49	0.21	9E−13	Age
cg13466600	X	77,587,444	NA	+	− 6.10	0.71	8E−18	− 3.63	0.46	5E−15	Age
cg05517106	X	135,286,461	*FHL1*	+	− 2.97	0.49	1E−09	− 2.43	0.36	2E−11	Age
cg11643285	3	16,411,667	*RFTN1*	+	− 0.09	0.01	2E−19	− 0.16	0.01	5E−122	Sex
cg09516963	12	68,042,445	*DYRK2*	–	− 0.06	0.01	8E−22	− 0.07	3.91E-3	2E−75	Sex
cg05100634	18	45,457,604	*SMAD2*	–	− 0.06	0.01	2E−30	− 0.03	4.46E-3	2E−11	Sex
cg10334916	2	241,508,098	*RNPEPL1*	+	0.03	0.01	2E−08	0.02	3.92E-3	2E−09	Sex
cg16532938	2	164,584,635	*FIGN*	–	− 0.12	0.02	1E−09	− 0.15	0.01	3E−22	Age-by-sex
cg06072257	1	11,434,636	NA	+	− 0.27	0.04	1E−11	− 0.33	0.04	3E−18	Age-by-sex
cg00531806	4	190,938,709	NA	–	− 0.12	0.02	4E−11	− 0.14	0.02	3E−16	Age-by-sex
cg15833111	X	49,166,019	*GAGE10*	+	0.22	0.02	1E−23	0.14	0.01	6E−27	Age-by-sex
cg05548968	X	30,928,416	NA	–	0.14	0.02	4E−14	0.08	0.01	5E−09	Age-by-sex
cg15475625	X	9,042,463	NA	–	0.21	0.03	1E−12	0.18	0.02	3E−14	Age-by-sex
cg20202246	X	118,407,296	NA	–	0.21	0.03	7E−13	0.23	0.03	1E−15	Age-by-sex
cg08814148	X	118,407,645	NA	+	0.12	0.02	5E−10	0.13	0.01	8E−18	Age-by-sex
cg24541420	X	135,691,028	NA	–	− 0.16	0.02	1E−15	− 0.12	0.01	4E−16	Age-by-sex

### DNAm and sex differences on the autosomes

There were 134,649 autosomal CpGs associated with sex at the epigenome-wide significant level (*p* < 3.6 × 10^−8^) in the discovery cohort; 69,384 were epigenome-wide significant with the effect sizes in the same direction in the replication cohort (Fig. 1; Additional file 1: Table S3). As with age, high genomic inflation was observed with lambda values ranging from 14.4 to 16.4 (Additional file 2: Figure. S3). The MLM approach that adjusted for inter-probe correlations and unmeasured confounders again showed much better control of genomic inflation (lambda values of 1.00 and 1.05; Additional file 2: Figure. S4). It identified 4 autosomal sites that were epigenome-wide significant with the effect sizes in the same direction in both the discovery and replication cohorts (Table 1). All of these sites were included in the 69,384 sites reported in Fig. 1 and Additional file 1: Table S3.

### DNAm and the interaction between chronological age and sex

The LR model that did not adjust for inter-probe correlations identified 85 autosomal and 635 X-linked CpGs that showed different ageing trajectories by sex (Additional file 1: Tables S4-S5; *p* < 3.6 × 10^−8^) in the discovery cohort. Fifty-two autosomal and 597 X-linked sites replicated (same direction and *p* < 3.6 × 10^−8^) in the replication cohort (Fig. 1). These mapped to 251 unique genes. Genomic inflation ranged from lambdas of 1.11 and 6.29 (Additional file 2: Figure. S5). A heterogeneity test between age EWAS outputs from sex-stratified models gave –log_10_ *p* values that were highly correlated with the –log_10_ *p* values from the interaction model (Additional file 2: Figure. S6).

The more conservative MLM approach (lambda range 0.94–1.00; Additional file 2: Figure. S7) identified four autosomal and seven X-linked sites that were epigenome-wide significant with the effect sizes in the same direction in both the discovery and replication cohorts (Additional file 1: Table S6). Nine of these sites were included in the 649 sites reported in Fig. 1 and Tables S4-S5.

**Fig. 2:**
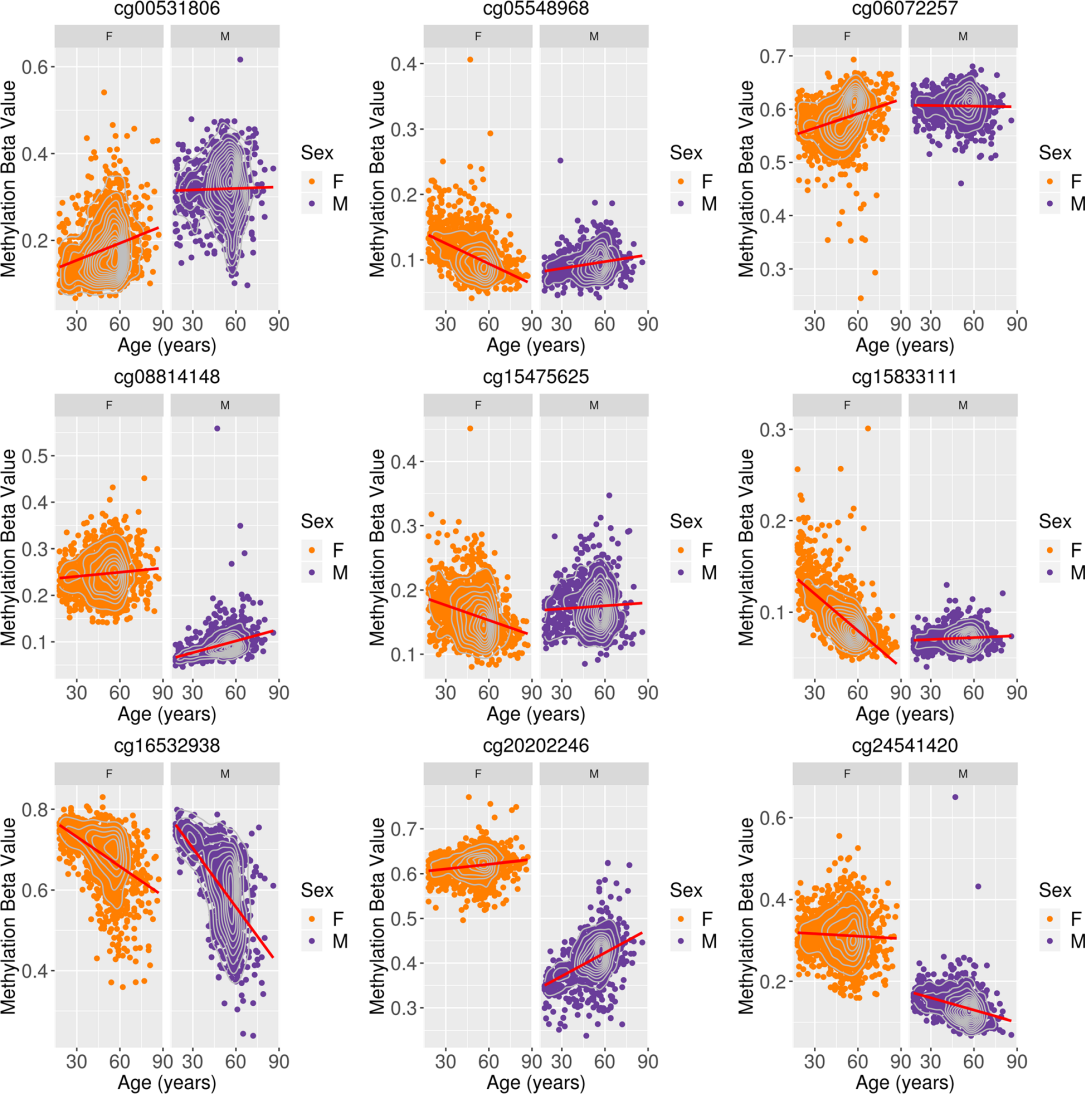
Pseudo-trajectories of DNA methylation by sex for probes associated with the age-by-sex interaction term using linear regression and MOMENT methods. DNA methylation beta values (*y*-axis) are plotted against chronological age (*x*-axis) separately for male samples (purple points) and female samples (orange points). Red lines represent the regression slope for the univariate model DNAm ~ age. Grey contour lines indicate data density

The probe with the greatest ageing-associated difference between males and females was cg15833111, mapping to *GAGE10* on the X chromosome (Fig. 2; interaction *p* < 1.41 × 10^−23^ in all LR and MLM models). In the discovery cohort, hypomethylation of this probe was observed with increasing age in females (*r* = − 0.61), whereas methylation levels remained stable in males (*r* = 0.02). Four of the 9 CpG sites identified as significant in both cohorts and by both modelling approaches exhibited clear differences in mean DNAm levels between males and females across an adult age range (cg00531806, cg08814148, cg20202246, cg24541420). These differences ranged from 0.14 to 0.21 on the beta value scale. There were significant main effects for both age and sex for 239 of the 649 probes (13 autosomal and 226 X-linked).

To investigate whether X chromosome inactivation (XCI) affected the 217 genes mapping to the 597 X-linked CpGs identified in this analysis, the genes were queried against a list of 114 XCI-escaping genes [25]. Thirty-two age-by-sex-associated genes were present in the list of 114 XCI escapees. There was an enrichment of XCI escapee genes in the list of age-by-sex-associated genes (*χ*^2^ = 4.19; *p* = 0.04) with 14.7% of the 217 age-by-sex associated genes being XCI escapees versus 9.6% in the remaining 850 genes (based on the EPIC array annotation) on the X chromosome.

**Fig. 3:**
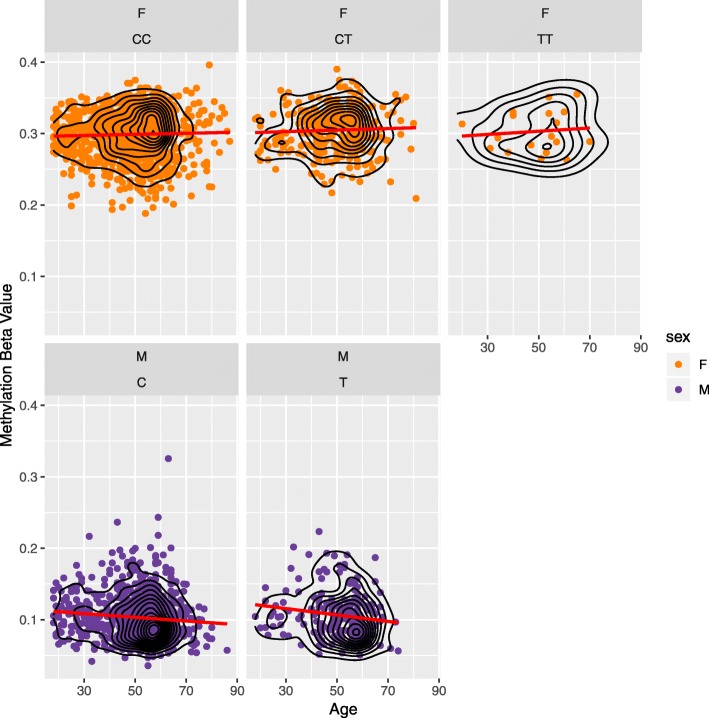
DNA methylation by genotype at the prostate cancer-associated variant rs17321482. DNA methylation beta values (*y*-axis) are plotted against chronological age (*x*-axis) separately for male samples (purple points) and female samples (orange points), based on genotype at rs17321482. Red lines represent the regression slope for the univariate model DNAm ~ age. Black contour lines indicate data density

Genes mapping to the 649 probes identified and replicated in the LR analysis were queried against published GWAS results (GWAS catalog[26]) to determine whether there was an enrichment among specific traits. Considering SNPs located within 1 Mb of a given age-by-sex-associated CpG site yielded 98 genome-wide significant associations (*p* < 5 × 10^−8^) comprising 73 SNPs (Additional file 1: Table S7) [27]. Several associations were with sexually dimorphic traits (e.g. prostate cancer, systemic lupus erythematosus, male pattern baldness, body mass index) [28–37]. All of the SNPs were located on the X chromosome. Of the 649 probes significantly associated with the age-sex interaction, 399 were also present on the Illumina 450K array (all of which were X-linked). These were queried against the ARIES methylation Quantitative Trait Locus (mQTL) database (mQTLdb [38];). There were 9664 CpG-SNP associations reported comprising 4582 unique SNPs and 279 unique CpGs (Additional file 1: Table S8). Two mQTL SNPs were present in the 73 SNPs identified from the GWAS catalogue query. These were associated with male pattern baldness and prostate cancer [31, 36]. The prostate cancer-associated SNP (rs17321482) is a QTL in an adolescent sample for the genome-wide significant age-by-sex CpG (cg23722529), mapping to *ARHGAP6*. Figure 3 shows a plot of methylation at cg23722529 by age, stratified by sex and genotype for rs17321482 in the discovery cohort. A simple linear regression model of CpG on the interaction between genotype and age suggested no association between DNA methylation and age in either sex (male interaction *P*_age_T_ = 0.40, female interaction *P*_age_CT_ = 0.86, *P*_*age*_TT_ = 0.82). Whereas ascertainment bias (e.g. sampling healthier older males or those that survive prostate cancer) may have been driving the association between cg23722529 and age in men, there was a minimal difference in the effect sizes in the whole population versus those aged under 60 years (*β*_all_ = − 0.0047, *p* = 1.2 × 10^−4^; *β*_< 60_ = − 0.0043, *p* = 5.5 × 10^−3^). The male pattern baldness SNP (rs79798752) maps to *EDA2R*, within ~ 554 kb from the EWAS CpG (cg15343840—an EPIC array-specific CpG) in the same gene. This SNP is a QTL for a different probe (cg08021299), approximately 249 kb away and mapping to *HEPH*. The probe is also among the 649 CpGs significantly associated with the age-by-sex interaction.

### Functional enrichment analysis

The 251 genes where differential methylation was associated with the age-by-sex interaction in the LR analysis were assessed for enrichment in KEGG pathways, GO terms, and tissue-specific expression in 30 general tissues from GTEx v7 [24, 39].

**Fig. 4:**
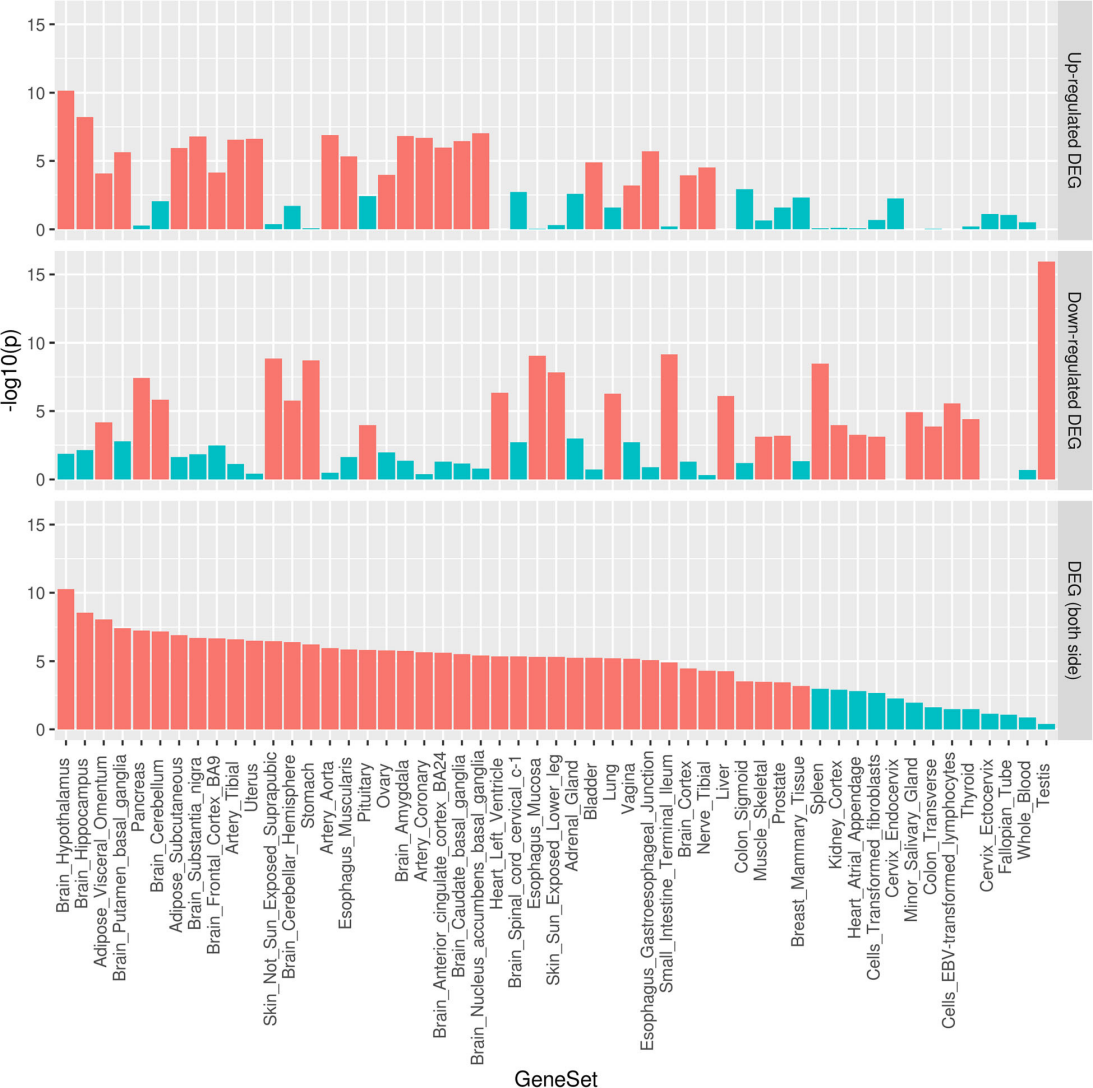
Enrichment of age-by-sex-associated genes among differentially expressed genes by tissue. The figure shows enrichment among differentially expressed genes in a given tissue (*x*-axis) compared to all other tissue types. The enrichment –log10(*p* value) is shown on the *y*-axis. Tissues with significant enrichment of genes are highlighted in red

There were no KEGG pathways or GO terms enriched for the genes associated with the age-by-sex interaction after correction for multiple testing (Bonferroni-adjusted *p* < 0.05; Additional file 1: Tables S9-S10). The strongest enrichment was observed among downregulated genes in the testis and upregulated genes in the brain regions including thy hypothalamus and hippocampus (Fig. 4).

## Discussion

Using a combination of standard linear regression methods and a more conservative mixed modelling method, we have identified loci displaying sexually dimorphic ageing associations in DNAm across the adult age range. These associations were predominantly X-linked. Of the nine CpGs identified by both methods, two mapped to genes (*GAGE10* and *FIGN*).

The site with the greatest absolute age-DNAm correlation difference between males and females mapped to *GAGE10*, a member of the *GAGE* cancer/testis antigen family [40]. Normal expression of GAGE proteins is limited to germ cells. However, *GAGE* transcripts have been observed in multiple cancers including melanomas and breast, lung, ovarian, and thyroid cancers [40–46].

The majority of age-by-sex-associated genes did not return results when queried in the GWAS catalogue. This is not surprising as the X chromosome is usually omitted from association studies [47]. However, of the associations identified, several pertained to sexually dimorphic traits. A variant in *ARGHAP6*, an age-by-sex-associated gene from this study, has previously been linked to prostate cancer [31]. However, there was no effect of this genotype on the age-by-sex interaction. Further examination of interactions between DNA methylation and disease-related genetic risk factors in a longitudinal context is warranted.

The mQTLdb resource is limited to probes present on the Infinium Human Methylation 450K BeadChip—the predecessor of the Infinium MethylationEPIC BeadChip used in the current study [38]. Of the 649 sites queried for mQTLs, 399 were present on both platforms. It is, therefore, possible that additional, as-yet-unidentified SNP associations are present among the remaining sites specific to the Infinium MethylationEPIC BeadChip.

Analysis of tissue-specific expression patterns of the genes displaying sexually dimorphic age-associated DNAm trajectories revealed strong enrichment among differentially expressed genes in the testis and brain regions, including the hypothalamus. Enrichment among differentially expressed genes in the testis may be indicative of a relationship with endocrine function and sex-specific ageing. This is consistent with the current hypotheses of endocrine differences as a contributor to the disparity in the life expectancy of males and females [48–50]. Additionally, the hypothalamus displays anatomical, ontogenetic, and biochemical differences between males and females [51]. The present findings may further delineate the established contribution of differential DNAm profiles to sex-specific disparities in the brain structure and function [52, 53].

In addition to the age-by-sex interaction, we examined the relationship between DNAm and both chronological age and sex. We replicated previous findings, with the strongest age-associated effects observed in *KLF14*, *ELOVL2*, and *FHL2* [14, 15] and sex-associated effects observed in *RFTN1* [54]. As replication was observed using both the liberal LR method and the more conservative MLM approach, these genes should be prioritised for further functional investigation in studies of ageing and sexually dimorphic traits.

The current study is strengthened by the use of large, unrelated discovery and replication cohorts with a broad age range, which has permitted the development of pseudo-longitudinal profiles of DNAm across the life course in males and females. An additional strength is the use of two modelling approaches. The more liberal linear regression approach does not account for correlations which may be present between probes and is prone to genomic inflation. However, in the current study, we accompanied our analysis with a more conservative mixed linear modelling approach (called MOMENT), which accounts for the relationship between a given probe and trait with distal probes fitted in multiple random effect components [22]. This approach can account for unobserved confounders and reduce inflation. However, this also poses a limitation in that results based on this method may be overly conservative. This is evidenced by the small number of age and sex associations identified using MOMENT.

## Conclusions

We identified 649 CpG sites displaying differences in age-associated DNAm patterns between males and females. The majority of these sites are located on the X chromosome, several of which are within genes associated with sexually dimorphic traits by GWAS, including prostate cancer and male pattern baldness. In order to identify the mechanisms of sex-specific differences in biological ageing, further investigation of sexually dimorphic characteristics of these processes over the life course is warranted.

## Supplementary information

**Additional file 1: Tables S1-S10:** Top 1000 autosomal probe associations for chronological age present in both discovery and replication cohorts, using the linear regression model, ranked by discovery *P*-value. Table headers correspond to Limma TopTable outputs - **Table S1.** Top 1000 X-linked probe associations for chronological age present in the discovery and replication cohorts, using the linear regression model, ranked by discovery P-value. Table headers correspond to Limma TopTable outputs - **Table S2.** Top 1000 autosomal probe associations for sex present in both discovery and replication cohorts, using the linear regression model, ranked by discovery P-value. Table headers correspond to Limma TopTable outputs - **Table S3.** Top 1000 autosomal probe associations for the age-by-sex interaction present in both discovery and replication cohorts, using the linear regression method, ranked by discovery P-value. Table headers correspond to Limma TopTable outputs - **Table S4.** Top 1000 X-linked probe associations for the age-by-sex interaction present in both discovery and replication cohorts, using the linear regression approach, ranked by discovery P-value. Table headers correspond to Limma TopTable outputs - **Table S5.** Epigenome-wide significant sites associated with the age-by-sex interaction with concordant effects in the discovery and replication cohorts identified using the conservative mixed-modelling approach - **Table S6.** GWAS catalogue outputs within 1 Mb of age-by-sex-associated CpGs - **Table S7.** ARIES mQTLdb output for query containing age-by-sex-associated CpGs - **Table S8.** KEGG pathway enrichment output for age-by-sex-associated sites - **Table S9.** GO Term enrichment output for age-by-sex-associated sites - **Table S10.**

**Additional file 2: Figures S1-S7. **Quantile-quantile plots for the discovery and replication EWASs of chronological age using the linear regression method - **Fig. S1.** Quantile-quantile plots for discovery and replication EWASs of chronological age using the conservative mixed-modelling method - **Fig. S2.** Quantile-quantile plots for the discovery and replication EWASs of sex using the linear regression method - **Fig. S3.** Quantile-quantile plots for discovery and replication EWASs of sex using the conservative mixed-modelling method - **Fig. S4.** Quantile-quantile plots for discovery and replication EWASs of the age-by-sex interaction using the linear regression method - **Fig. S5.** Heterogeneity test *P*-values for male-only EWAS of age versus female-only EWAS of chronological age in the discovery and replication sets - **Fig. S6.** Quantile-quantile plots for discovery and replication EWASs of the age-by-sex interaction using the conservative mixed-modelling method - **Fig. S7.**

## Data Availability

According to the terms of consent for GS participants, access to individual-level data (omics and phenotypes) must be reviewed by the GS Access Committee. Applications should be made to access@generationscotland.org. Full summary statistics for the analyses presented are publicly available online at 10.7488/ds/2709.
