# Supplementary material for: An epigenome-wide association study of sex-specific chronological ageing
Source: Genome Med. 2019 Dec 31;12:1. doi: 10.1186/s13073-019-0693-z (PMC6938636; doi:10.1186/s13073-019-0693-z)
Supplement: Supplementary file 2 — Additional file 2: Figures S1-S7. Quantile-quantile plots for the discovery and replication EWASs of chronological age using the linear regression method - Fig. S1. Quantile-quantile plots for discovery and replication EWASs of chronological age using the conservative mixed-modelling method - Fig. S2. Quantile-quantile plots for the discovery and replication EWASs of sex using the linear regression method - Fig. S3. Quantile-quantile plots for discovery and replication EWASs of sex using the conservative mixed-modelling method - Fig. S4. Quantile-quantile plots for discovery and replication EWASs of the age-by-sex interaction using the linear regression method - Fig. S5. Heterogeneity test P-values for male-only EWAS of age versus female-only EWAS of chronological age in the discovery and replication sets - Fig. S6. Quantile-quantile plots for discovery and replication EWASs of the age-by-sex interaction using the conservative mixed-modelling method - Fig. S7. [file 13073_2019_693_MOESM2_ESM.pdf]

**Fig. S1: Age EWAS QQ plots (LR method)**

Observed –log10(p-values) on the y-axis are plotted against theoretical –log10(p-values) on the x-axis. Upper panels correspond to the discovery data whereas lower panels correspond to the replication data. Autosomal (left panels) and X-chromosomal probes (right panels) were analysed separately.

**Fig. S2: Age EWAS QQ plots (MLM method)**

Observed –log10(p-values) on the y-axis are plotted against theoretical –log10(p-values) on the x-axis. Upper panels correspond to the discovery data whereas lower panels correspond to the replication data. Autosomal (left panels) and X-chromosomal probes (right panels) were analysed separately.

**Fig. S3: Sex EWAS QQ plots (Autosomal probes, LR method)**

Observed –log10(p-values) on the y-axis are plotted against theoretical –log10(p-values) on the x-axis. Upper panels correspond to the discovery data whereas lower panels correspond to the replication data.

**Fig. S4: Sex EWAS QQ plots (Autosomal probes, MLM method)**

Observed –log10(p-values) on the y-axis are plotted against theoretical –log10(p-values) on the x-axis. Upper panels correspond to the discovery data whereas lower panels correspond to the replication data.

**Fig. S5: Age-by-Sex EWAS QQ plots (LR method)**

Observed –log10(p-values) on the y-axis are plotted against theoretical –log10(p-values) on the x-axis. Upper panels correspond to the discovery data whereas lower panels correspond to the replication data. Autosomal (left panels) and X-chromosomal probes (right panels) were analysed separately.

**Fig. S6: Heterogeneity Test p-values vs Age-by-Sex p-values:**

Heterogeneity test of p-values for male EWAS of age vs female EWAS of age (x-axes) are plotted against age-by-sex EWAS p-values (y-axes). P-values from the discovery set are presented in the upper panels and the replication set p-values are presented in the lower panels. Autosomal and x-chromosomal probes were analysed separately (left and right panels, respectively).

**
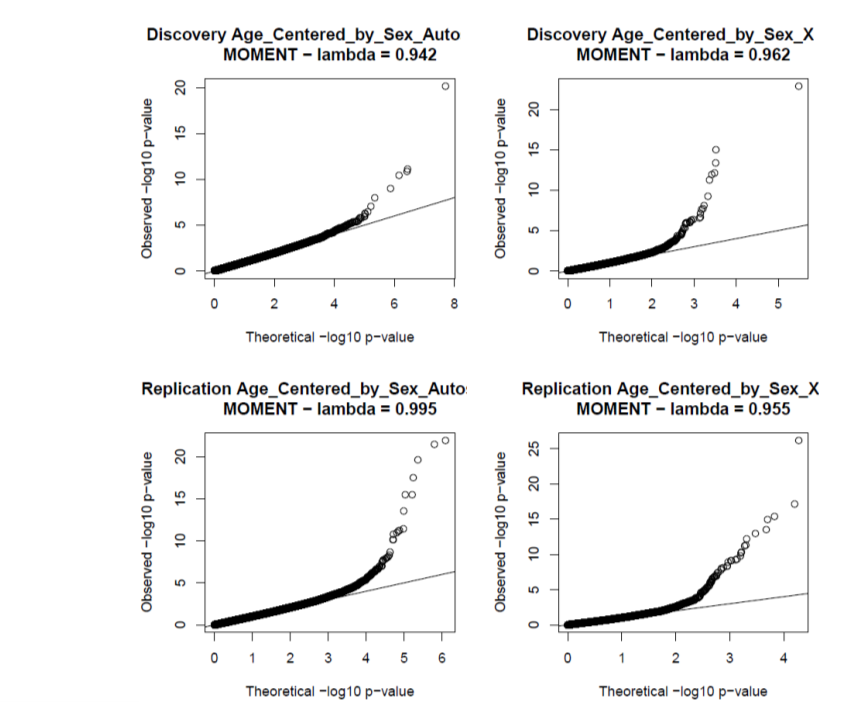
**

**Fig. S7: Age-by-Sex EWAS QQ plots (MLM method)**

Observed –log10(p-values) on the y-axis are plotted against theoretical –log10(p-values) on the x-axis. Upper panels correspond to the discovery data whereas lower panels correspond to the replication data. Autosomal (left panels) and X-chromosomal probes (right panels) were analysed separately.
